# Efficacy and safety of venetoclax plus hypomethylating agents in relapsed/refractory acute myeloid leukemia: a multicenter real-life experience

**DOI:** 10.3389/fonc.2024.1370405

**Published:** 2024-04-12

**Authors:** Francesco Angotzi, Federica Lessi, Matteo Leoncin, Carla Filì, Mauro Endri, Albana Lico, Andrea Visentin, Stefano Pravato, Anna Candoni, Livio Trentin, Carmela Gurrieri

**Affiliations:** ^1^ Hematology Unit, Azienda Ospedale-Università and University of Padova, Padua, Italy; ^2^ Hematology Unit, Azienda Ulss3 Serenissima, Ospedale dell’Angelo, Venice, Italy; ^3^ Division of Hematology and Bone Marrow Transplantation, Azienda Sanitaria Universitaria Integrata Friuli Centrale (ASUFC), Udine, Italy; ^4^ Hematology Section, Dipartimento di Medicina Specialistica, Ca’ Foncello Hospital, Treviso, Italy; ^5^ Hematology and Cell Therapy Division, San Bortolo Hospital, Vicenza, Italy

**Keywords:** acute myeloid leukemia, venetoclax, hypomethylating agents, azacitidine, decitabine, relapsed/refractory acute myeloid leukemia

## Abstract

Venetoclax (VEN) has been shown to play a synergistic effect in combination with hypomethylating agents (HMAs) in the frontline treatment of acute myeloid leukemia (AML). However, the potential role of this therapy in the relapsed/refractory (R/R) AML setting, still needs to be further unveiled. The aim of the current study was to retrospectively outline the safety profile, response and survival outcomes of R/R AML patients treated with VEN in association with HMAs. Clinical, biological, and molecular data were collected from 57 patients with R/R AML treated with VEN combined with azacitidine or decitabine between 2018 and 2023. The median age of patients was 63 years, 38 (66.7%) received treatment for relapsed disease while 19 (33.3%) for refractory disease, 5 (8.7%) were treated for molecular relapse. A consistent proportion of the cohort was represented by patients with unfavorable prognostic factors such as complex karyotype (36.8%), secondary AML (29.8%), previous exposure to HMAs (38.6%), and relapse after allogeneic stem cell transplant (22.8%). A total of 14 patients achieved CR (24.6%), 3 (5.3%) CRi, 3 (5.3%) MLFS, and 3 (5.3%) PR, accounting for an ORR of 40.4%. The CR/CRi rate was higher in the group treated with azacitidine than in the group treated with decitabine (37.8% vs. 15%). The median OS was 8.2 months, reaching 20.1 months among responding patients. VEN-HMAs treatment allowed to bridge to allogeneic stem cell transplantation 11 (23.9%) of eligible patients, for which a median OS of 19.8 months was shown. On multivariate analysis, ECOG performance status ≥2, complex karyotype and not proceeding to allogeneic stem cell transplantation after therapy with VEN-HMAs were the factors independently associated with shorter OS. Patients treated with the azacitidine rather than the decitabine containing regimen generally displayed a trend toward superior outcomes. The major toxicities were prolonged neutropenia and infections. In conclusion, this study showed how VEN-HMAs could represent an effective salvage therapy in patients with R/R AML, even among some of those patients harboring dismal prognostic features, with a good toxicity profile. Further prospective studies are thus warranted.

## 1 Introduction

Despite novel therapies and the curative potential of allogeneic hematopoietic stem cell transplantation (allo-HSCT) that have progressively improved the outcomes of patients with acute myeloid leukemia (AML) (1), relapse occurs in more than one half of patients and is the primary cause of death. Also, around 20% of patients are primary refractory to the induction regimen. As the prognosis of relapsed/refractory (R/R) patients to remains poor, with an estimated 5-year overall survival (OS) of almost 10% (2, 3), the availability of novel therapies represents an important unmet clinical need. Salvage multi-agent chemotherapy regimens achieve poor results in this setting, complete remission (CR) rates are in the range of 20 – 65% and response durations are short, lasting less than 1 year (3), thus limiting their usefulness to patients who are eligible to subsequent allo-HSCT consolidation. The hypomethylating agents (HMAs) azacitidine (AZA) and decitabine (DEC) administered as single agents are also employed in the R/R setting (4). However, results with these agents are also disappointing, with a response rate of 16.3% and a median OS of 6.7 months reported in a large multicenter study (4). Newer targeted agents showed somewhat more encouraging results, often superior to those obtained by conventional treatments. In the ADMIRAL trial, the FLT3 inhibitor gilteritinib performed better than salvage chemotherapy in R/R AML patients (median OS 9.3 months vs. 5.6 months in the gilteritinib and chemotherapy groups respectively) (5), and the IDH1/2 inhibitors ivosidenib and enasidenib demonstrated encouraging results as well (6, 7). At the present time, allo-HSCT remains the only therapeutic option for long-term disease control in R/R patients, resulting in 3 to 5 year OS of 15-25% (3, 8, 9).

Venetoclax (VEN) is an orally administered BH3 mimetic that blocks the anti-apoptotic B-cell lymphoma 2 protein, inducing apoptosis in cancer cells (10). Its introduction has changed the treatment landscape of chronic lymphocytic leukemia, and it has gained therapeutic niches in non-Hodgkin lymphoma and multiple myeloma (10, 11). In AML, after the first evidence that demonstrated significant antileukemic activity of VEN as single agent (12), this drug has been mostly studied in combination therapies with different agents in both treatment naïve and R/R patients (13). The pivotal VIALE-A phase III trial subsequently tested the combination of VEN-AZA versus AZA alone in newly diagnosed patients considered unfit for standard induction chemotherapy. The combination of VEN with AZA resulted in both higher response rates (composite complete remission 66.4% vs. 28.3%) and significantly prolonged OS (median 14.7 months vs. 9.6 months) (14). Based on these results, this combination has become a standard of care for newly diagnosed AML patients unfit for induction chemotherapy. Although a direct comparison in a randomized phase III trial is lacking, the association of VEN with DEC showed superiority when compared to DEC monotherapy in a propensity score-matched analysis, yielding an estimated median OS of 13.4 months vs. 8.3 months, and response rates of 70.3% vs. 24.3% for VEN-DEC and DEC respectively (15). These results unveiled the attractive possibility of testing VEN in association with HMAs in R/R AML. Indeed, several studies, although mostly retrospective and with limited numbers of patients, have reported a good activity of VEN-HMAs based combinations in patients with R/R AML (16). In the present study, we aim to provide further evidence on the efficacy and safety of this combination in R/R AML by reporting our multicenter experience.

## 2 Patients and methods

### 2.1 Study design and outcome measures

The medical records of R/R AML patients treated with VEN-HMAs from five medical institutions in Italy were retrospectively reviewed. Relevant information regarding baseline patients’ characteristics, previous treatment lines, AML type (de novo/secondary), cytogenetics, molecular data, and therapy with VEN-HMAs were recorded in an anonymous database. Information regarding treatment-emergent adverse events (AEs) during therapy with VEN-HMAs was also collected. Only patients with complete safety data were included in the final safety evaluation, AEs were classified according to the Common Terminology Criteria for Adverse Events (CTCAE) version 5.0.

All included patients were diagnosed with R/R AML and received at least one line of therapy. Patients who previously received either single-agent VEN or VEN combined with other agents such as low-dose cytarabine were not included in the study. All clinical, cytogenetic, and molecular data were recorded at the time of relapse before the initiation of VEN-based therapy. AML was classified according the 2022 International Consensus Classification (ICC) (17). Refractoriness was defined as being unable to achieve at least a partial remission (PR) after at least two cycles of intensive induction chemotherapy. Response criteria and outcome measures were evaluated according to the 2022 European Leukemia Net (ELN) response criteria, including OS, event-free survival (EFS), relapse-free survival (RFS), and cumulative incidence of relapse (CIR) (18). Objective response rate (ORR) was defined as a composite outcome of complete remission (CR), complete remission with incomplete hematologic recovery (CRi), partial remission (PR), and morphologic leukemia-free state (MLFS). Relapse-free survival (RFS) and the cumulative incidence of relapse (CIR) were calculated only for patients achieving a CR or CRi.

After a short ramp-up dose from day 1 to day 3, VEN was administered at the dose of 400 mg/day, or 100 mg/day in patients with concomitant use of azole antifungal prophylaxis, continuously from days 1 to 28 of each treatment cycle of 28 days. AZA was administered by subcutaneous injection at the dose of 75 mg/m^2^ from day 1 to 7 and DEC by intravenous infusion at the dose of 20 mg/m^2^ from day 1 to 5 of each treatment cycle.

This study was performed in agreement with the Declaration of Helsinki and approved by the relative institutions research ethics committees (number:1430/A3). Informed consent was obtained from all patients.

### 2.2 Statistical analysis

Population characteristics were summarized using descriptive statistics. Difference between groups that received AZA or DEC associated with VEN were analyzed by Fisher’s exact test or Mann-Whitney U test as appropriate. Response rates were compared using the Chi-square test. Median follow-up time was calculated by the revers Kaplan-Meier estimator. OS, EFS and RFS were estimated by the Kaplan-Meier estimator and survival curves were compared using the log-rank test. The CIR was estimated by the cumulative incidence function. Variables associated with objective response were investigated by univariate and multivariate logistic regression. The association of variables with OS and EFS was explored by univariate and multivariate Cox Proportional Hazard models. Significant variables in univariate analysis were included in multivariate models. All p-values are two-sided, with a significance level of 0.05. Statistical analyses were conducted with R software (version 4.1.3).

## 3 Results

### 3.1 Population characteristics

A total of 57 patients were included in the study, 28 (49.1%) females and 29 (50.9%) males. The median age was 63 years (range: 20 – 80; IQR: 17). Before the start of treatment, the reported ECOG performance status (ECOG PS) was ≥2 in 33% of patients, those treated with the VEN-DEC combination had more frequently an ECOG PS of 2-4 (55% vs. 21.6% in the VEN-AZA group, p = 0.02). At the time of relapse or confirmation of refractory disease, 21 (36.9%) patients harbored AML with complex karyotype and 26 (45.6%) AML with normal karyotype. Other cytogenetical abnormalities including +8, +9, del (7), t (2,12), t (2,3), t (9,11) and r (19) were reported in 9 (15.8%) patients. According to the ICC 2022 classification, 24 (42.1%) patients were classified as having AML with myelodysplasia related gene mutations/cytogenetics, 14 (24.6%) AML with mutated NPM1, 3 (5.3%) AML with mutated TP53, 1 (1.8%) AML with t (9, 11)(p21.3;q23.3)/MLL::KMT2A, and 15 (26.3%) AML not otherwise specified. Diagnosis of de-novo AML and secondary AML was made in 40 (70.2%) and 17 (29.8%) patients respectively. Diagnosis of therapy-related AML was made in 5 (8.8%) patients, who were previously treated with chemotherapy for breast cancer, ovarian cancer, non-Hodgkin’s lymphoma or multiple myeloma. A total of 32 (56.1%) patients received VEN-HMAs treatment for relapsed disease while 25 (43.9%) for disease refractory to the last line of therapy. The median number of prior therapies was 1 (range 1 – 3) (not including a second induction regimen), and 20 (35.1%) patients received ≥2 lines of therapy. A previous exposure to HMAs was reported in 22 patients (38.6%). Of those, 13 (59.1%) and 5 (22.7%) patients received AZA or DEC respectively for the treatment of AML, while the remaining 4 (18.2%) received AZA for MDS. A total of 5 (8.7%) patients were treated with VEN-HMAs after molecular relapse, and they all received AZA as and HMA. 22 (38.6%) patients were previously exposed to HMAs, and 13 (22.8%) relapsed after a previous allo-HSCT. A total of 37 (65%) patients were treated with AZA and 20 (35%) with DEC in combination with VEN. The groups treated with VEN-AZA and VEN-DEC significantly differed only in terms of ECOG PS distribution. Complete patients’ demographics and characteristics are illustrated in **Table 1**.

**Table 1:** Baseline population’s characteristics.

	All patients (n=57)	VEN-AZA (n=37)	VEN-DEC (n=20)	P-values
Age, years (%)				
Median (range)	63 (20–80)	62 (23–80)	64 (20–77)	0.20
<65	34 (59.6)	24 (64.9)	10 (50)	
65 - 75	18 (31.6)	12 (32.4)	6 (30)	
>75	5 (8.8)	1 (2.7)	4 (20)	
**Sex (%)**				0.58
Female	28 (49.1)	17 (45.9)	11 (55)	
Male	29 (50.9)	20 (54.1)	9 (45)	
**ECOG PS**				**0.02**
0 – 1	34 (59.6)	26 (70.3)	8 (40)	
2 – 4	19 (33.3)	8 (21.6)	11 (55)	
Not available	4 (7.1)	3 (8.1)	1 (5)	
Karyotype				
Complex	21 (36.8)	12 (32.4)	9 (45)	0.68
Normal	26 (45.6)	18 (48.6)	8 (40)	
Other	9 (15.8)	6 (16.2)	3 (15)	
Not available	1 (1.8)	1 (2.7)	0	
NPM1 status				
Mutated	14 (24.6)	9 (24.3)	5 (25)	1
Wild type	39 (68.4)	25 (67.6)	14 (70)	
Not available	4 (7.0)	3 (8.1)	1 (5)	
FLT3 status				
Mutated	9 (15.8)	6 (16.2)	3 (15)	1
Wild type	44 (77.2)	28 (75.7)	16 (80)	
Not available	4 (7.0)	3 (8.1)	1 (5)	
IDH-1/2 status				
Mutated	8 (14.0)	5 (13.5)	3 (15)	1
Wild type	43 (75.4)	27 (73.0)	16 (80)	
Not available	6 (10.5)	5 (13.5)	1 (5)	
Type				
De novo	40 (70.2)	27 (73)	13 (65)	0.75
Secondary	17 (29.8)	10 (27)	7 (35)	
**Therapy-related**	5 (8.8)	2 (5.4)	3 (15)	0.33
ICC 2022				
AML-MR	24 (42.1)	16 (43.2)	8 (40)	0.75
AML-NPM1	14 (24.6)	9 (24.3)	5 (25)	
AML-TP53	3 (5.3)	1 (2.7)	2 (10)	
AML-t(9;11)	1 (1.8)	1 (2.7)	0	
AML-NOS	15 (26.3)	10 (27.0)	5 (25)	
**Relapsed**	32 (56.1)	21 (56.8)	11 (55)	1
**Refractory**	25 (43.9)	16 (43.2)	9 (45)	1
**Previous lines of therapy, median (range)**	1 (1 – 3)	1 (1 – 3)	1 (1 – 3)	0.06
Previous treatment type				
3+7	20 (35.1)	16 (43.2)	4 (20)	0.98
3+7+GO	2 (3.5)	0 (0)	2 (10)	
3+7+Midostaurin	7 (12.5)	5 (13.5)	2 (10)	
CPX-351	6 (10.5)	4 (10.8)	2 (10)	
FLAI	26 (45.6)	17 (45.9)	9 (45)	
AZA	13 (22.8)	11 (29.7)	2 (10)	
DEC	5 (8.8)	2 (5.4)	3 (15)	
ICE	4 (7)	3 (8.1)	1 (5)	
MEC	8 (14)	4 (10.8)	4 (20)	
DAV	1 (1.8)	1 (2.7)	0 (0)	
HAM	2 (3.5)	1 (2.7)	1 (5)	
CLA	1 (1.8)	1 (2.7)	0 (0)	
Sorafenib	2 (3.5)	1 (2.7)	1 (5)	
Gilteritinib	4 (7)	4 (10.8)	0 (0)	
CAR-NK	1 (1.8)	1 (2.7)	0 (0)	
**Previous exposure to HMAs**	22 (38.6)	16 (43.2)	6 (30)	0.48
**Previous allo-HSCT**	13 (22.8)	9 (24.3)	4 (20)	1

FLAI, Fludarabine-Cytarabine-Idarubicin; ICE, Idarubicin-Cytarabine-Etoposide; MEC, Mitoxantrone-Etoposide-Cytarabine; DAV, Daunorubicin-Cytarabine-Etoposide; HAM, Cytarabine-Mitoxantrone; CLA, Clofarabine-Cytarabine.

Bold values indicate significant P-values

### 3.2 Response evaluation

**Figure 1:**
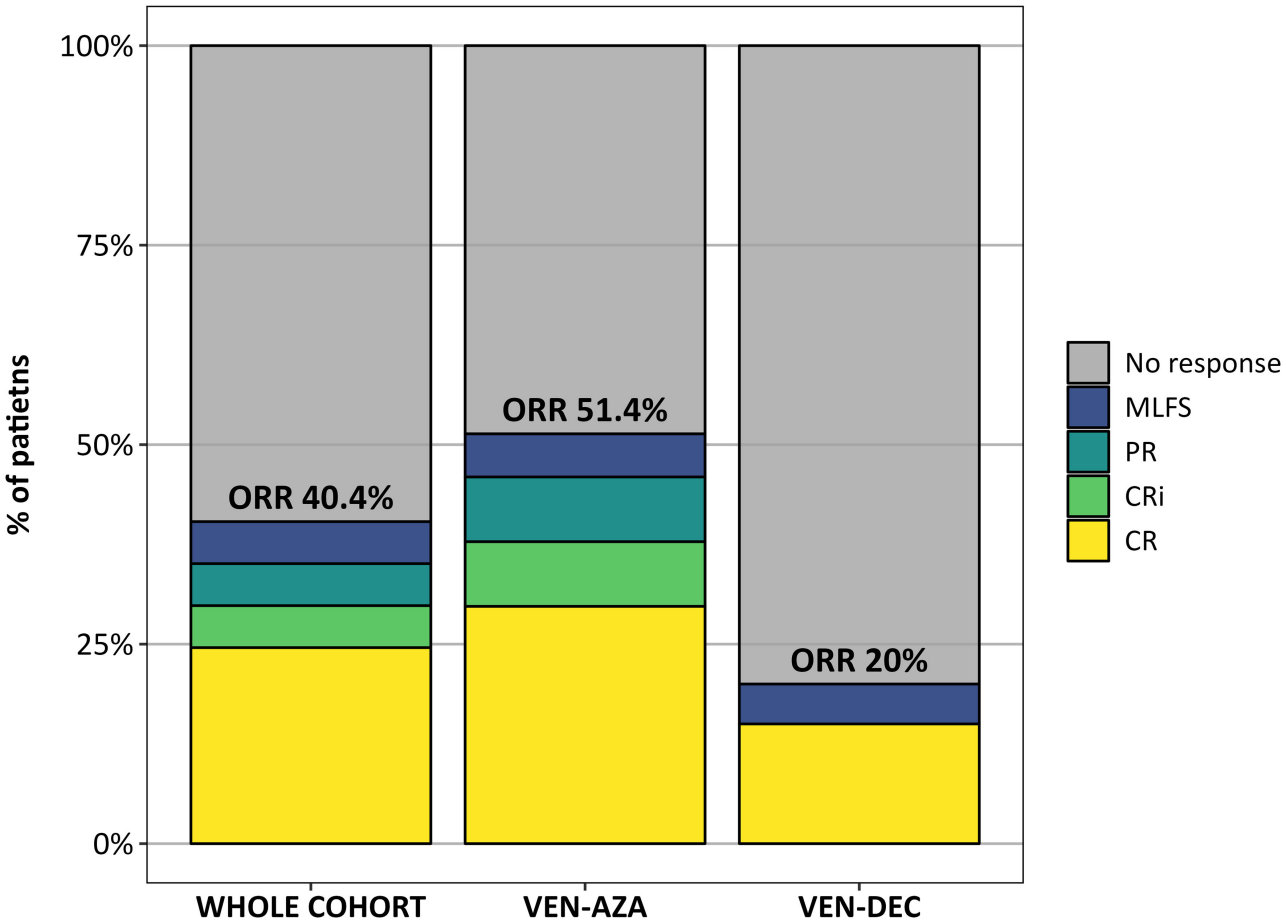
Response rates in the whole cohort and in patients treated with VEN-AZA or VEN-DEC.

All patients received at least one cycle of therapy with VEN combined with AZA or DEC between January 2018 and January 2023. The median number of treatment cycles was 3 (range 1 – 14) in the VEN-AZA group and 2 (range 1 – 8) in the VEN-DEC group. In the whole cohort, a total of 14 (24.6%) patients achieved a CR, 3 (5.3%) a CRi, 3 (5.3%) a MLFS, and another 3 (5.3%) a PR, accounting for an ORR of 40.4%. There was a trend towards a higher composite complete remission rate (CR/CRi) in the group treated with AZA versus the group treated with DEC (37.8% vs. 15%; p = 0.13), and consequently a significantly higher ORR in the VEN-AZA group versus the VEN-DEC group (51.4% vs. 20%; p = 0.04) (**Figure 1**). Responses were seen in 17/35 (48.6%) patients not previously exposed to HMAs and in 6/22 (27.3%) in patients previously exposed to HMAs (p = 0.18). Eleven out of 46 eligible patients underwent allo-HSCT after therapy with VEN-HMAs (23.9%), 8 (72.7%) after VEN-AZA and 3 (27.3%) after VEN-DEC. Patients who proceeded to allo-HSCT did not receive further cycles with VEN-HMAs after transplant. Of these, 9 patients obtained an objective response with VEN-HMAs and were bridged directly to allo-HSCT, while the 3 other patients required further salvage therapy before transplantation. The remaining 35 allo-HSCT eligible patients were not bridged to allo-HSCT due to disease progression, and 6 (13%) discontinued VEN due to toxicity. Among patients who received only 1 previous line of therapy, the ORR was 43.2% vs. 35% in those who received ≥ 2 lines, resulting in a non-statistically significant difference in ORR (p = 0.75).

### 3.3 Survival outcomes

**Figure 2:**
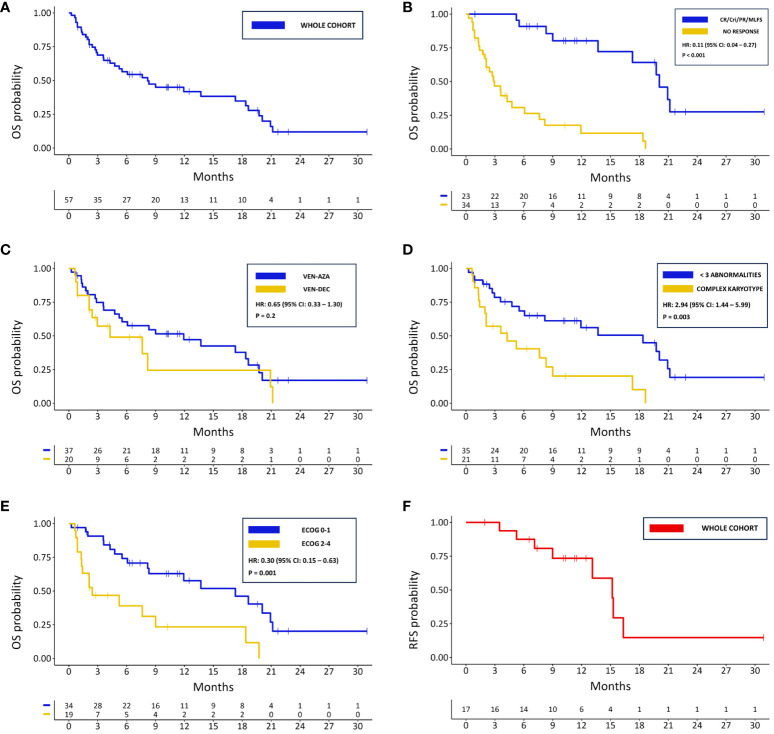
Kaplan-Meier survival curves for: **(A)** OS in the whole cohort; **(B)** OS in responding and not responding patients; **(C)** OS based on HMA associated with VEN; **(D)** OS for patients with complex karyotype and <3 cytogenetical abnormalities; **(E)** OS based of ECOG PS; **(F)** PFS in the whole cohort.

After a median follow-up time of 19.6 months (IQR: 15.41), 20 (35.1%) patients were still alive. The median OS in the whole cohort was 8.2 months (95% CI: 4.76 – 18.60) (**Figure 2A**), and the median EFS of 2.45 months (95% CI: 1.97 – 7.33). A longer median OS was observed in patients achieving at least a MLFS compared to those who did not (20.07 months vs. 2.96 months; HR: 0.11; 95% CI: 0.04 – 0.27; p < 0.001) (**Figure 2B**). There was a non-statistically significant trend towards a longer OS in patients treated with VEN-AZA compared to those threated with VEN-DEC (median OS 11.93 vs. 4.27 months; HR: 0.65; 95% CI: 0.33 – 1.30; p = 0.2) (**Figure 2C**). A similar tendency was observed regarding EFS (median 2.86 vs. 2.00 months for the VEN-AZA and VEN-DEC groups respectively; HR: 0.65; 95% CI: 0.33 – 1.27; p = 0.2). Significant differences in OS were also found between patients harboring complex karyotype or not (median 4.3 vs. 18.4 months; HR: 2.94; 95% CI: 1.44 – 5.99; p = 0.003) (**Figure 2D**) and between those having an ECOG PS 0-1 or 2-4 (median 17.3 vs. 2.43 months; HR: 0.30; 95% CI: 0.15 – 0.63; p = 0.001) (**Figure 2E**). Patients that underwent therapy with VEN-HMAs after a relapse from allo-HSCT had a median OS of 11.93 months (95% CI: 2.96 – NR) and a median EFS of 2 months (95% CI: 2.00 – NR). Although patients that did not previously receive an allo-HSCT had a shorter median OS of 7.62 months (95% CI: 4.27 – 18.6), this difference was not statistically significant (HR: 0.75; 95% CI: 0.31 – 1.81; p = 0.5). No difference in OS and EFS was found between patients who previously received 1 line of therapy versus those who received >1 (median OS 7.62 vs. 11.93 months, HR: 0.91, 95% CI: 0.47 – 1.81, p = 0.8; median EFS 2.86 vs. 2.00 months, HR: 0.79, 95% CI: 0.41 – 1.52, p = 0.5). Among patients achieving a CR/CRi, a total of 7 (41.2%) patients eventually relapsed, showing a median RFS of 15.2 months (**Figure 2F**) and an estimated CIR at 12 months of 19% (95% CI: 4.4 – 42). Among the 10 patients who underwent allo-HSCT after VEN-HMAs, the median OS was 19.75 months (95% CI: 17.28 – NR), being significantly longer than those who did not proceed to allo-HSCT (19.75 vs. 5.22 months; HR: 0.31; 95% CI: 0.11 – 0.90; p = 0.01). Among the patients that were treated for molecular relapse, 3 out of 5 achieved a CR with undetectable MRD status, while the other 2 did not show a response. Their overall survival ranged from 18.6 to 5.52 months, only one patient eventually progressed to allo-HSCT, and 2 remained alive.

### 3.4 Predictors of response and survival

In univariate analysis, higher age and secondary AML were associated with a lower probability of obtaining an objective response, while an ECOG PS ≤1 and therapy with VEN-AZA were significantly associated with a higher probability of achieving objective response (**Table 2**). In multivariate analysis, only ECOG PS ≤1 and secondary AML remained significantly associated with response (**Table 3**). Regarding OS, in univariate Cox regression an ECOG PS ≤1 was associated longer survival, and complex karyotype and secondary AML with shorter OS (**Table 4**). All these three variables were also independently associated with OS in multivariate analysis (**Table 5**). Univariate and multivariate results for EFS are represented in **Supplementary Tables 1**, **2**. Notably, ECOG PS and complex karyotype were significantly associated with longer and shorter EFS respectively in univariate analysis, but not in multivariate analysis (**Supplementary Tables 1**, **2**).

**Table 2:** Univariate logistic regression results for variables associated with objective response (CR/CRi/PR/MLFS).

Variable	OR	95% CI	P values
Age	**0.95**	**0.90 – 0.99**	0.03
Sex (M vs. F)	0.81	0.28 – 2.37	0.71
ECOG PS (≤ 1 vs. ≥ 2)	**7.62**	**2.07 – 37.41**	**0.005**
Karyotype (Complex vs. not)	0.42	0.16 – 1.30	0.14
NPM1 (Mutated vs. WT)	2.13	0.62 – 7.68	0.23
FLT3 (Mutated vs. WT)	1.81	0.42 – 8.20	0.42
TP53 (Mutated vs. WT)	0.92	0.10 – 8.71	0.94
IDH 1/2 (Mutated vs. WT)	2.31	0.50 – 12.48	0.29
Secondary vs. de novo	**0.21**	**0.04 – 0.78**	0.03
Therapy related vs. not	NA	0.00 – ∞	0.99
Relapsed vs. refractory	1.86	0.64 – 5.76	0.26
Previous exposure to HMAs	0.40	0.12 – 1.21	0.16
Previous allo-HSCT	1.36	0.38 – 4.79	0.63
Previous lines of therapy (1 vs. ≥ 2)	1.41	0.47 – 4.53	0.55
VEN-AZA vs. VEN-DEC	**4.22**	**1.27 – 16.96**	0.03

Bold values indicate significant P-values

NA, Not applicable.

**Table 3:** Multivariate logistic regression results for variables associated with objective response (CR/CRi/PR/MLFS).

Variable	OR	95% CI	P values
Age	0.94	0.88 – 1.00	0.06
ECOG PS (≤ 1 *vs*. ≥ 2)	**12.42**	**2.35 – 106.90**	**0.007**
Secondary vs. de novo	**0.18**	**0.03 – 0.88**	**0.045**
VEN-AZA vs. VEN-DEC	3.09	0.68 – 16.01	0.15

Bold values indicate significant P-values

**Table 4:** Univariate Cox proportional hazards model for OS.

Variable	HR	95% CI for HR	P values
Age	1.02	0.99 – 1.05	0.11
Sex (M vs. F)	0.89	0.46 – 1.73	0.74
ECOG PS (≤ 1 vs. ≥ 2)	**0.30**	**0.15 – 0.63**	**0.001**
Karyotype (Complex vs. not)	**2.94**	**1.44 – 5.99**	**0.003**
NPM1 (Mutated vs. WT)	0.93	0.42 – 2.07	0.86
FLT3 (Mutated vs. WT)	1.06	0.40 – 2.79	0.91
TP53 (Mutated vs. WT)	0.57	0.10 – 4.40	0.59
IDH 1/2 (Mutated vs. WT)	0.86	0.35 – 2.14	0.75
Secondary vs. de novo	**2.64**	**1.32 – 5.27**	**0.006**
Therapy related vs. not	2.16	0.83 – 5.64	0.12
Relapsed vs. refractory	0.61	0.32 – 1.17	0.14
Previous exposure to HMAs	1.19	0.62 – 2.29	0.60
Previous allo-HSCT	0.75	0.31 – 1.81	0.53
Previous lines of therapy (1 vs. ≥ 2)	0.92	0.47 – 1.81	0.80
VEN-AZA vs. VEN-DEC	0.65	0.33 – 1.30	0.22
Allo-HSCT after VEN-HMAs	**0.31**	**0.11 – 0.90**	**0.03**

Bold values indicate significant P-values

**Table 5:** Multivariate Cox proportional hazards model for OS.

Variable	HR	95% CI for HR	P values
ECOG PS (≤1 vs. ≥2)	**0.28**	**0.13 – 0.60**	**0.001**
Karyotype (Complex vs. not)	**2.51**	**1.17 – 5.39**	**0.018**
Secondary vs. de novo	2.06	0.97– 4.41	0.061
Allo-HSCT after VEN-HMAs	**0.32**	**0.11 – 0.94**	**0.038**

Bold values indicate significant P-values

### 3.5 Safety

Complete safety data were reported for 44/57 (77.2%) patients, which were thus included in the safety analysis. Overall, cytopenias were the most common AEs and were mostly severe. A total of 95.4% of patients experienced grade 3-4 neutropenia, 72.7% grade 3-4 thrombocytopenia, and 45.5% grade 3-4 anemia (**Supplementary Table 3**). 28 patients experienced at least an episode of febrile neutropenia (FN), accounting for 63.6% of the patients included in the safety analysis, and for a total of 50 episodes of FN recorded during therapy with VEN-HMAs. Of all patients experiencing FN, in 23 (82.1%) it was graded as G3, and in 4 as G4 (14.3%). One episode of FN resulted in death, which was the only fatal AE recorded (**Supplementary Table 3**). The most common minor (grade ≤2) AEs were anemia (22.6%), constipation (9.1%), and fatigue (9.1%) (**Supplementary Table 4**). No episodes of tumor lysis syndrome were reported. Six early deaths (<30 days from the start of therapy) occurred in the whole cohort, yet they were all due to failure to respond to therapy and subsequent disease progression.

## 4 Discussion

Salvage therapy in R/R AML still represents a major unmet clinical need. While therapy with VEN-HMAs has been established as the standard of care in the frontline treatment of unfit AML patients, evidence regarding its efficacy in the R/R setting is still needed. Response rates and survival outcomes in R/R AML treated with VEN-HMAs described in the literature are quite heterogeneous. Reported ORR ranged from 21% to 64%, CR/CRi rates from 12% to 61%, and median OS from 3 to 10.7 months (19–36) (**Supplementary Table 5**). In our study, combination treatment with VEN-HMAs led to an ORR in 40.4% and a CR/CRi rate of 29.8%. While non-responding patients experienced a very poor outcome with median OS around 3 months, response to treatment translated into favorable survival rates of around 20 months and allowed to bridge to allo-HSCT almost 20% of patients. These results are noteworthy in a cohort enriched with patients carrying dismal prognostic factors, such as complex karyotype, secondary AML, previous treatment with HMAs or relapse post allo-HSCT.

Subgroup analysis showed a trend towards superior outcomes in patients treated with VEN-AZA rather than VEN-DEC. This was particularly true regarding ORR, with exposure to VEN-AZA being associated with higher chances of obtaining an objective response in univariate analysis. This difference however must be interpreted with caution. First of all, it could have resulted from bias induced by the low number of patients treated with VEN-DEC included in the analysis. Secondly, the worse outcomes observed with VEN-DEC may be due to a larger portion of patients in this subgroup carrying an ECOG PS ≥2 which has been consistently associated with lower rates of response and shorter OS in both univariate and multivariate analyses. While AZA and DEC have previously demonstrated comparable efficacy when administered as single agents (37–39), inferior results with the combination VEN-DEC were previously reported in a series of R/R patients treated with VEN-HMAs. Here, therapy with VEN-AZA also resulted in a trend towards longer OS compared to VEN-DEC (25 months vs. 5.4 months; p = 0.13) (31). One reason for the poor performance of VEN-DEC observed in our analysis might have also been the 5-day schedule employed for DEC, since pharmacodynamic evidence suggests that a 10-day schedule, by increasing the exposure of AML blasts to DEC, may produce high response rates as shown in a phase I and a phase II trial in R/R AML (40–42). Noteworthy, as reported in a retrospective study by Maiti et. al., despite high response rates (ORR 60%), survival outcomes where not particularly encouraging with the 10-day schedule (median OS 6.8 months and median EFS 5.7 months) (35). Thus, the superiority of the longer schedule is still debatable, especially in R/R AML, and direct comparisons are necessary.

Response rates were found to be higher in patients who previously received only one line of therapy, and while this did not translate into a survival advantage, it may still highlight the importance of employing VEN-HMAs earlier as a salvage treatment, considering both the likelihood of developing progressive sub-clonal complexity with the related onset of therapy resistance, and increasing patient unfitness as the disease progresses (43). Prior therapy with HMAs and prior allo-HSCT, which conventionally predict poor response to subsequent therapies, did not preclude, even to a lesser extent, a response to treatment or worse survival. This finding is corroborated by other reports (21, 36), and supports, in the absence of clinical trials, the use of VEN-HMAs in this historically poor risk patients subsets. However, it is also possible that prior allo-HSCT did not influence prognosis due to bias induced by low numbers, and due to the fact that patients included in this cohort were already heavily pretreated and carrying an unfavorable prognosis independently of previous allo-HSCT. Even though patients who previously underwent allo-HSCT had a longer median OS than those who did not, this difference was not statistically significant, and we believe it to be purely numerical, supporting the efficacy of VEN-HMAs independently of previous allo-HSCT. Secondary AML was universally associated with lower response rates and shorter OS in our analysis. This is not unexpected as secondary AML more frequently harbors poor risk genetic features and patients are generally older (36, 44, 45). This appeared even more true in our experience with R/R patients, which highlights the possibility of observing lower outcomes with VEN-HMAs combinations in R/R secondary AML.

While complex karyotype carried a worse prognosis regarding OS and EFS, we could not find significant molecular predictors of response or survival. This is likely due to the low number of events that hindered the emergence of significant associations. Yet, it must be noted that other studies found some degree of association with such variables. Trends toward better responses and longer survival were identified in patients with IDH 1/2, NPM1, SRSF2, and RUNX1 mutations in three studies (22, 30, 32). On the other side, TP53, KRAS/NRAS, DNMT3A, and SF3B1 have generally been associated with worse outcomes (22, 32). Instead, the role of FLT3 mutations remains elusive, with conflicting results reported in the literature (22, 30).

The longer OS observed in patients who subsequently underwent allo-HSCT refrains the fact that stem cell transplantation after the achievement of a CR/CRi is still the only viable, and possibly curative, option for patients with R/R AML. It is in this context that VEN-HMAs combinations may play a role in R/R AML, by providing a line of treatment that can act as a bridge to allo-HSCT. Another study has specifically evaluated the use of VEN-AZA for this particular purpose, and its results were comparable to ours. In this report, 6 out of 10 patients achieved a response and subsequently underwent allo-HSCT, with a median OS of 11.7 months in transplanted patients (46). Very similar results to ours were also observed in the larger AVALON study were 25.9% of R/R patients were bridged to allo-HSCT which resulted in a OS benefit (36).

An intriguing clinical scenario may be the use of VEN-HMAs in a pre-emptive fashion in patients with AML who manifest molecular relapse with increasing MRD levels. In this setting, there is already evidence for the efficacy of single agent AZA. The RELAZA-2 phase II trial explored the use of AZA to prevent or delay hematological relapse in MRD-positive patients, who achieved 12-months OS and EFS rates of 94% and 44% (47). In those who eventually relapsed, hematologic relapse was delayed by a median of 10.6 months (48). Outcomes adopting the same pre-emptive strategy, but utilizing VEN-based combination regimens, were reported in a small study including 10 patients, where 50% achieved negative MRD status (49). Our results are in line with these studies and suggest this as another strategy worth exploring in future studies.

An interesting point coming from our experience is the failure of VEN-HMAs to provide a satisfactory OS/EFS benefit for patients with poor ECOG performance status. Thus, while VEN-AZA use in the first line setting has shown remarkable results in patients unfit for intensive chemotherapy in the VIALE-A trial, the benefit of this combination in older, unfit R/R AML patients should be thoroughly evaluated, as this therapy may fail to improve survival while at the same time increasing the rate of AEs, in a group of patients for whom preserving quality of life is a priority. Although age is also commonly associated with worse OS in AML, we could not demonstrate its adverse prognostic impact on survival in either univariate or multivariate analysis, likely due to the low number of participants in the study.

Regarding safety, as expected the most common adverse events were cytopenias, which were experienced by virtually all patients. Overall, more than half of patients experienced at least one episode of FN. These results are in line with those shown by other studies that reported safety outcomes of VEN-HMAs in R/R AML, with similar rates of adverse events and FN, the latter ranging from 40% to 83.3% (22–25, 27, 29–31, 33). Afterall, given the absence of early deaths due to therapy and of only one fatal AE, the VEN-HMAs combination proved to be relatively safe in our cohort.

In conclusion, our analysis adds to the body of evidence exploring the use of VEN-HMAs in R/R AML, while waiting for the results of prospective clinical trials that are currently ongoing (NCT03404193, NCT04905810, NCT05362942). Like other studies on this topic, its retrospective nature and the low patient numbers pose significant limitations. Systematic reviews and meta-analyses may only partially fill this knowledge gap, allowing us to synthesize the findings of numerous retrospective experiences and to overcome some of the drawbacks associated with having multiple reports each containing low patient numbers. What may be most needed, however, are prospective trials comparing VEN-HMAs with HMAs alone, intensive chemotherapy, or other targeted agents in the R/R AML setting. These studies should provide guidance on how to identify the R/R AML patients who are most likely to benefit from treatment with VEN-HMAs. Nonetheless, VEN-HMAs should be regarded as another therapeutic option able to induce remission in R/R AML, which are worth a try in such an aggressive and unforgiving disease.

## Data availability statement

The raw data supporting the conclusions of this article will be made available by the authors, without undue reservation.

## Ethics statement

The studies involving humans were approved by Azienda ospedale-università Padova (REV-AL01). The studies were conducted in accordance with the local legislation and institutional requirements. The participants provided their written informed consent to participate in this study.

## Author contributions

FA: Data curation, Formal analysis, Methodology, Validation, Visualization, Writing – original draft, Writing – review & editing. FL: Conceptualization, Supervision, Writing – review & editing. ML: Methodology, Writing – review & editing. CF: Writing – review & editing. ME: Writing – review & editing. AL: Writing – review & editing. AV: Methodology, Supervision, Validation, Writing – review & editing. SP: Supervision, Writing – review & editing. AC: Conceptualization, Methodology, Supervision, Visualization, Writing – review & editing. LT: Writing – review & editing. CG: Conceptualization, Data curation, Investigation, Methodology, Project administration, Supervision, Writing – review & editing.
