# Supplementary material for: Efficacy and safety of venetoclax plus hypomethylating agents in relapsed/refractory acute myeloid leukemia: a multicenter real-life experience
Source: Front Oncol. 2024 Apr 12;14:1370405. doi: 10.3389/fonc.2024.1370405 (PMC11045980; doi:10.3389/fonc.2024.1370405)
Supplement: Supplementary file 1 [file Table_1.docx]

Supplementary Material

**Supplementary table 1:** Univariate Cox proportional hazards model for EFS

| **Variable** | **HR** | **95% CI for HR** | **P values** |
| --- | --- | --- | --- |
| Age | 1.01 | 0.98 – 1.04 | 0.50 |
| Sex (M vs. F) | 0.94 | 0.50 – 1.79 | 0.86 |
| ECOG PS (≤1 vs. ≥2) | **0.22** | **0.10 – 0.47** | **< 0.001** |
| Karyotype (Complex vs. not) | **2.76** | **1.40 – 5.51** | **0.004** |
| NPM1 (Mutated vs. WT) | 0.60 | 0.27 – 1.32 | 0.20 |
| FLT3 (Mutated vs. WT) | 0.81 | 0.33 – 1.96 | 0.64 |
| TP53 (Mutated vs. WT) | 2.51 | 0.78 – 8.05 | 0.12 |
| IDH 1/2 (Mutated vs. WT) | 0.81 | 0.33 – 2.00 | 0.65 |
| Secondary vs. de novo | 1.50 | 0.71 – 3.17 | 0.28 |
| Therapy related vs. not | 1.27 | 0.39 – 4.18 | 0.6 as |
| Relapsed vs. refractory | 0.26 | 0.13 – 1.52 | 0.23 |
| Previous exposure to HMAs | 0.94 | 0.48 – 1.84 | 0.86 |
| Previous allo-HSCT | 0.73 | 0.32 – 1.66 | 0.45 |
| Previous lines of therapy (1 vs. ≥2) | 0.79 | 0.41 – 1.52 | 0.49 |
| VEN-AZA vs. VEN-DEC | 0.65 | 0.33 – 1.27 | 0.21 |
| Allo-HSCT after VEN-HMAs | 0.84 | 0.38 – 1.84 | 0.66 |

**Supplementary table 2:** Multivariate Cox proportional hazards model for EFS

| **Variable** | **HR** | **95% CI for HR** | **P values** |
| --- | --- | --- | --- |
| ECOG PS (≤1 vs. ≥2) | 0.47 | 0.20 – 1.11 | 0.08 |
| Karyotype (Complex vs. not) | 1.86 | 0.89 – 3.90 | 0.10 |

**Supplementary table 3:** Major adverse events (grade ≥ 3)

| **Adverse event** | **G3 (%)** | **G4 (%)** | **G5 (%)** | **Total (%)** |
| --- | --- | --- | --- | --- |
| Neutropenia | 3 (6.8) | 39 (88.6) | 0 | 42 (95.4) |
| Thrombocytopenia | 4 (9.1) | 28 (63.6) | 0 | 32 (72.7) |
| Anemia | 16 (36.4) | 4 (9.1) | 0 | 20 (45.5) |
| Febrile neutropenia | 23 (52.3) | 4 (9.1) | 1 (2.2) | 28 (63.6) |
| Sepsis | 1 (2.2) | 3 (6.8) | 0 | 4 (9.1) |
| Pneumonia | 1 (2.2) | 1 (2.2) | 0 | 2 (4.5) |
| Perianal abscess | 1 (2.2) | 0 | 0 | 1(2.2) |
| Abdominal infection | 1 (2.2) | 0 | 0 | 1 (2.2) |
| Soft tissue infection | 1 (2.2) | 0 | 0 | 1 (2.2) |
| Enterocolitis | 0 | 1 (2.2) | 0 | 1 (2.2) |
| Atrial fibrillation | 2 (4.5) | 0 | 0 | 2 (4.5) |
| Constipation | 1 (2.2) | 0 | 0 | 1 (2.2) |
| Syncope | 1 (2.2) | 0 | 0 | 1 (2.2) |
| Fatigue | 2 (4.5) | 0 | 0 | 2 (4.5) |

**Supplementary table 4:** Minor adverse events (grade ≤ 2)

| **Adverse event** | **G1 (%)** | **G2 (%)** | **Total (%)** |
| --- | --- | --- | --- |
| Neutropenia | 0 | 1 (2.2) | 1 (2.2) |
| Thrombocytopenia | 1 (2.2) | 1 (2.2) | 2 (4.5) |
| Anemia | 1 (2.2) | 9 (20.4) | 10 (22.6) |
| Diarrhea | 1 (2.2) | 2 (4.5) | 3 (6.8) |
| Constipation | 0 | 4 (9.1) | 4 (9.1) |
| Nausea | 1 (2.2) | 2 (4.5) | 3 (6.8) |
| Vomiting | 1 (2.2) | 0 | 1 (2.2) |
| Oral mucositis | 3 (6.8) | 0 | 3 (6.8) |
| Fatigue | 0 | 4 (9.1) | 4 (9.1) |
| Hypokalemia | 1 (2.2) | 2 (4.5) | 3 (6.8) |
| Hyponatremia | 0 | 1 (2.2) | 1 (2.2) |
| Dyspnea | 1 (2.2) | 1 (2.2) | 2 (4.5) |
| Hypoxia | 0 | 1 (2.2) | 1 (2.2) |
| Oral hemorrhage | 1 (2.2) | 0 | 1 (2.2) |
| Deep venous thrombosis | 0 | 1 (2.2) | 1 (2.2) |
| Peripheral edema | 0 | 1 (2.2) | 1 (2.2) |
| Increased ALT/AST | 2 (4.5) | 0 | 2 (4.5) |
| Pruritus | 1 (2.2) | 1 (2.2) | 2 (4.5) |
| Maculo-papular rash | 0 | 1 (2.2) | 1 (2.2) |
| Muscle cramps | 0 | 1 (2.2) | 1 (2.2) |
| Pneumonia | 0 | 1 (2.2) | 1 (2.2) |
| HSV reactivation | 0 | 2 (4.5) | 2 (4.5) |
| Penile infection | 0 | 1 (2.2) | 1 (2.2) |
| Pharyngitis | 0 | 1 (2.2) | 1 (2.2) |

**Supplementary table 5:** Summary of studies and observed outcomes in patients with R/R AML treated with VEN-HMAs

| **Study (ref. n.)** | **Patients n.** | **ORR** | **CCR** | **OS** | **Other survival outcomes** |
| --- | --- | --- | --- | --- | --- |
| Di Nardo et al.(19) | 43*^†^ (31 VEN-HMAs) | 26%^†^* | 16.1%^†^* | Median 3.0 months^†^* | NR |
| Aldoss et al.(20) | 33 | 64% | 51% | 1-year OS 53% | Median LFS 8.9 months |
| Aldoss et al.(21) | 90 | NR | 46% | Median 7.8 months | Median LFS 8.9 months |
| Wang et al.(22) | 40* (21 VEN-HMAs) | 50%* | 22.5%* | Median 6.6 months* | NR |
| Piccini et al.(23) | 47* (34 VEN-HMAs) | NR | 55%* | Median 10.7 months* | Median EFS 4.5 months* |
| Byrne et al.(24) | 16* (12 VEN-HMAs) | 80% | 41.7% | Median 7.8 months* | NR |
| Joshi et al.(25) | 29 | 38% | 28% | Median 2.9 months | Median LFS 8.5 months |
| Goldberg et al.(26) | 24* (8 VEN-HMAs) | 28.6%* | 23.8%* | 3-month OS 72%* | NR |
| Feld et al.(27) | 39 | NR | 38.5% | Median 8.1 months | Median DOR 8.5 months |
| Ganzel et al.(28) | 40* (25 VEN-HMAs) | NR | 52%* | Median 5.5 months* | NR |
| Gaut et al.(29) | 14* (13 VEN-HMAs) | 35.7%* | 21.4%* | Median 4.7 months* | NR |
| Lou et al.(30) | 48 | 47.9% | 47.9% | Median 9.6 months | NR |
| Morsia et al.(31) | 44 | 42.9% | 33.3% | Median 5 months | Median PFS not reached Median DOR 6 months |
| Stahl et al.(32) | 86* (55 VEN-HMAs) | 40% | 30.9% | Median 25 months (VEN-AZA) Median 5.4 months (VEN-DEC) | Median DOR 10.2 months (VEN-AZA) Median DOR NR  (VEN-DEC) |
| Tenold et al.(33) | 25 | 52% | 32% | Median 5.5 months | Median DOR 14.7 months |
| Tong et al.(34) | 22 | 45.5% | 40.9% | Median 6 months | NR |
| Maiti et al.(35)^±^ | 65 | 60% | 42% | Median 6.8 months | Median EFS 5.7 months Median RFS 8.4 months |
| Todisco et al.(36) | 147 | 46.3% | 36.1% | Median 12.7 months (Refractory patients) Median 6.3 months  (Relapsed patients) | Median EFS 6.2 months (Refractory patients) Median EFS 4.4 months  (Relapsed patients) |

*Includes patients treated with VEN monotherapy or VEN associated with low dose cytarabine
†Includes patients treated for myelodysplastic syndrome or BPDCN (Blastic plasmacytoid dendritic-cell neoplasm)
±VEN administered with 10-day DEC schedule in the whole cohort
NR: not reported; ORR: overall response rate; CCR: composite complete remission rate; OS: overall survival; LFS: leukemia-free survival; EFS: event-free survival; RFS: relapse-free survival; DOR: duration of response.
